# Prevalence and influencing factors of subjects at high risk for cardiovascular disease in different regions of Gansu province, China: a cross-sectional study of 100,725 residents from 2017 to 2022

**DOI:** 10.3389/fcvm.2024.1373123

**Published:** 2024-08-05

**Authors:** Yixuan Li, Chouji Zhang, Faqing Chen, Jing Zhang

**Affiliations:** Department of Community Health and Chronic Non-Communicable Disease Control, Gansu Provincial Center for Disease Prevention and Control, Lanzhou, China

**Keywords:** cardiovascular disease, high-risk subjects, prevalence rate, influencing factors, different regions

## Abstract

**Background:**

To investigate the prevalence rate of subjects at high risk for cardiovascular disease (CVD) and to analyze the influencing factors in different regions of Gansu Province.

**Methods:**

We used data from the China Patient-centered Evaluative Assessment of Cardiac Events (PEACE) Million Persons Project (MPP), which screened 100,725 residents aged 35–75 years from 10 project sites in Gansu Province, China, from 2017 to 2022. In addition, a questionnaire survey, anthropometric measurements, and collection of biological samples were carried out.

**Results:**

Of the 100,082 residents included, 21,059 were identified as subjects at high-risk for CVD. The overall prevalence rate of subjects at high risk for CVD was 19.7%, and the prevalence rate in the HeXi region was greater than that in the LongZhong and LongDong regions. The prevalence rates were 14.0%, 58.2%, 34.9%, and 5.7% for cardiovascular history, hypertension, dyslipidemia, and WHO-assessed risk ≥20%, respectively. The prevalence rate of cardiovascular history type was the highest in the HeXi region, hypertension and dyslipidemia types were the highest in the LongZhong region, and WHO-assessed risk ≥20% type was the highest in the LongDong region. Male, higher education level, smoking status, snoring status, overweight and obesity status, central obesity status, and disease history were more likely to be risk factors for subjects at high risk for CVD. There were some differences among different regions in age, annual household income, farming status, rural/urban status, and drinking status.

**Conclusion:**

The prevalence rate of subjects at high risk for CVD in Gansu Province is relatively high. Individualized intervention measures as well as comprehensive prevention and control strategies should be adopted, focusing on the distribution characteristics of risk factors among high-risk subjects in different regions.

## 1 Introduction

Cardiovascular disease (CVD) is the main cause of morbidity and mortality worldwide. In 2017, CVD caused an estimated 17.8 million deaths worldwide, corresponding to 330 million years of life lost and another 35.6 million years lived with disability (1, 2). The burden of disease caused by CVD has been gradually increasing in China, and CVD caused almost 4 million deaths in 2016 (3). Nearly 80% of global CVD deaths occur in low- and middle-income countries (4). Gansu Province is located in northwestern China and has well-developed animal husbandry, agriculture, and mining industries, ranking third in the northwestern region (5). However, its per capita income and educational level are not high, its gross domestic product (GDP) ranked 27th in China in 2022 (5), and its primary medical and health resources are insufficient (6). Due to the geographical characteristics of Gansu Province, residents in different regions often consume strongly flavored foods such as pork, beef, and lamb, which are high in salt, fat, and calories, as well as drink for warding off cold temperatures (7). Therefore, investigating the prevalence and influencing factors of CVD in Gansu Province has epidemiological value.

Early identification of high-risk individuals is a crucial strategy in the primary prevention of CVD, based on the availability of appropriate CVD risk assessment models and guideline recommendations (8). Improving the main risk factors that can be changed is the current goal of CVD control (9). In addition, it is also crucial to identify the differences in the distribution of the main risk factors and the clustering of high-risk types of CVD and how these are associated with different regions. However, for CVD risk in Gansu Province, previous studies have included small sample sizes, have focused on a narrow spectrum of risk factors, or have not distinguished between different regions (10).

To fill these knowledge gaps, the aim of the present study was to investigate the prevalence rate and influencing factors of subjects at high risk for CVD in different regions of Gansu Province. The findings of this study can serve as a reference for CVD prevention in Gansu Province, China.

## 2 Materials and methods

### 2.1 Data sources and study populations

A government-funded public health program was conducted in Gansu Province, which is part of the China Patient-centered Evaluative Assessment of Cardiac Events (PEACE) Million Persons Project (MPP), which was launched by the China National Center for Cardiovascular Diseases (NCCD) (11). The project consists of the three following stages: (1) screening and recruitment, (2) a short-term (3 months) follow-up, and (3) a long-term (12 months) follow-up, with the objective of identifying populations with high risk of CVD and investigating their treatment, management, and outcomes. The screening subjects met the following criteria: (1) aged 35–75 years, (2) lived in the selected regions for at least 6 of the previous 12 months, and (3) voluntarily participated and signed an informed consent form (11, 12). The project collected data from face-to-face questionnaire interviews, physical examinations, and laboratory measurements (11).

**Figure 1:**
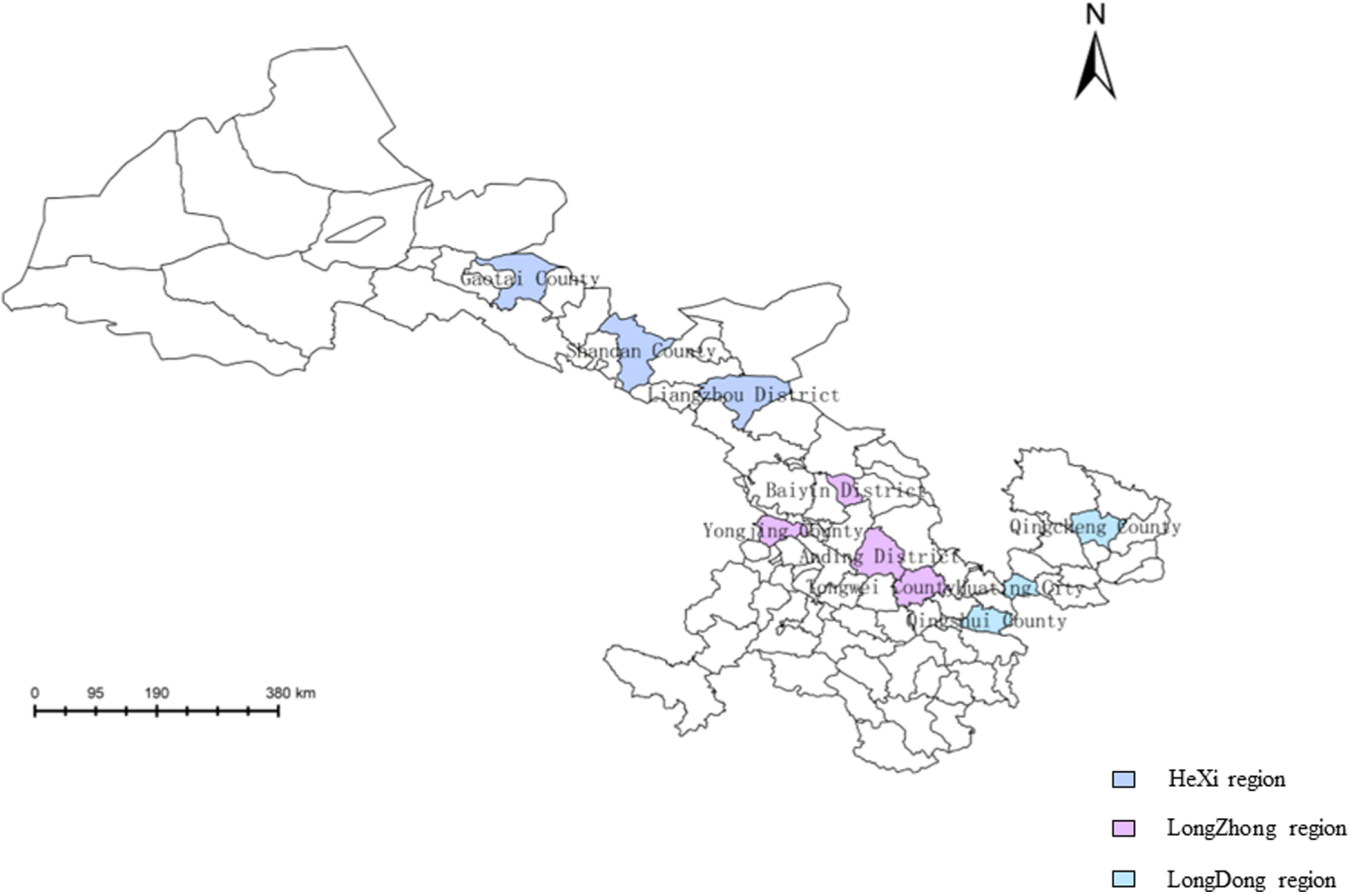
Map of all counties and districts of Gansu Province (including the 10 project sites).

There were eight project sites in 2017–2021, and two new project sites were added in 2022, for a total of 10 project sites. From 2017 to 2022, 100,725 permanent residents aged 35–75 years from the 10 project sites in Gansu Province, China, were recruited. According to the human geographical location, the project sites in Gansu Province were divided into the HeXi region, LongZhong region, and LongDong region (Figure 1) (13). The total population of Gansu Province in 2023 was 24.6 million. The percentages of the population enrolled in this study in the HeXi, LongDong, and LongZhong regions were 28.7%, 35.1%, and 36.2%, respectively. Subjects who had data missing with regard to age, annual household income, education level, or body mass index (BMI) were excluded. Finally, 100,082 participants were recruited and screened, and 21,059 high-risk subjects for CVD were identified. The study was approved by the Central Ethics Committee of the China National Center for Cardiovascular Disease, Beijing, China (Approval No. 2014–574). All enrolled participants provided written informed consent.

### 2.2 Data collection and measurement

Anthropometric measurements included height, weight, and waist circumference (WC). Participants were required to wear light clothes, with no shoes and no headgear, while height and weight were measured. BMI was calculated as weight (kg) divided by height squared (m^2^). Weight status based on BMI and WC was defined according to the Chinese standard (14). BMI was classified as underweight (<18.5), normal weight (18.5–23.9), overweight (24.0–27.9), or obese (≥28.0). Central obesity was defined as a WC ≥ 90 cm in men and ≥85 cm in women.

Seated blood pressure was measured twice on the right upper arm after 5 min of rest with an automatic digital sphygmomanometer. If the difference between the two systolic blood pressure (SBP) and diastolic blood pressure (DBP) readings were greater than 10 mmHg, a third measurement was obtained, and the average of the last two readings was used. A fasting vein blood glucose test was performed with a rapid glucose analyzer, and total cholesterol (TC), triglyceride (TG), high-density lipoprotein cholesterol (HDL-C), and low-density lipoprotein cholesterol (LDL-C) levels were measured with a rapid lipid analyzer.

### 2.3 Outcome variables

Participants at high risk for CVD were defined as meeting one of the following criteria based on the Chinese PEACE protocol (11, 15): (1) had a history of any disease or treatment of myocardial infarction (MI), ischemic or hemorrhagic stroke, percutaneous coronary intervention (PCI), or coronary artery bypass graft (CABG); (2) had SBP ≥ 160 mmHg or DBP ≥ 100 mmHg; (3) had LDL-C ≥ 4.14 mmol/L or HDL-C < 0.78 mmol/L; and (4) according to the Risk Prediction Chart of the Guidelines for Cardiovascular Risk Assessment and Management, the World Health Organization (WHO) determined a 10-year risk of CVD ≥ 20% based on age, gender, SBP, current smoking status (or who quit smoking for less than 1 year before the assessment), diabetes status (previously diagnosed diabetes, consumed hypoglycemic drugs, or injected insulin), and TC.

After all the investigation results were entered into the information collection system, the system automatically determined whether the screening subjects were at high risk for CVD, and the high-risk subjects were divided into four types based on the Chinese PEACE protocol: (1) cardiovascular history type, (2) hypertension type, (3) dyslipidemia type, and (4) WHO-assessed risk ≥20% type (11, 15).

### 2.4 Exposure variables

Sociodemographic and lifestyle factors data were collected using a structured questionnaire. The subjects were categorized into the five following age groups: 35–39, 40–49, 50–59, 60–69, and 70–75 years. Educational attainment was divided into primary school and below, junior high school, high school/technical secondary school, and college degree or above. Annual household income was divided into >50,000 and ≤50, 000. Occupation (farmer/non-farmer), gender (male/female), and areas (urban/rural) were also considered.

In addition, smoking status, drinking status, and snoring status were categorized into yes and no. We also collected participants’ disease history (yes or no), including hypertension, diabetes, dyslipidemia, and self-reported cardiovascular and cerebrovascular history.

### 2.5 Statistical analysis

First, we used a multivariate test of Little's Missing Completely at Random (MCAR) to verify the missing data were completely missing at random, and it indicated that the missing data were independent of the variable's value, therefore, we removed the missing data. We examined the basic characteristics and risk factors for subjects at high risk for CVD according to region. We tested the differences by region using Chi-square tests or Wilcoxon rank-sum tests when appropriate.

Age- and gender-adjusted prevalence rates of each high-risk type in subjects at high risk for CVD were calculated for overall and population subgroups (e.g., by region, age, gender, educational attainment, household income, and weight status) using the direct method based on the 2020 Chinese population census.

Mixed effects models were used to further explore the risk factors associated with subjects at high risk for CVD in different regions. All models were adjusted for age, gender, occupation, smoking status, drinking status, annual household income, educational attainment, BMI, central obesity, areas, and disease history. The effect sizes are presented as odds ratios (OR) and 95% confidence intervals (CIs).

Analyses were conducted using Stata 15.0 (StataCorp, College Station, TX, USA) and ArcGIS 10.5 (Esri). Statistical significance was set at *P* < 0.05.

## 3 Results

### 3.1 The demographic characteristics of subjects at high risk for CVD

Of the total subjects at high risk for CVD from 2017 to 2022, the HeXi region, LongZhong region, and LongDong region had 6,203, 7,544, and 7,302 subjects with high risk for CVD, respectively.

The proportion of subjects aged 60–69 years was greater in the HeXi (37.0%) and LongZhong regions (34.3%), while the proportion of subjects aged 50–59 years was greater in the LongDong region (38.7%). The subjects at high risk for CVD in the HeXi region had a higher proportion of males (52.4% vs. 49.9% and 48.1%) and non-farmers (53.5% vs. 38.6% and 22.8%) than in other regions. Compared with the subjects at high risk for CVD in the HeXi and LongZhong regions, the LongDong region had a higher percentage of subjects who completed primary school and below (68.0% vs. 45.2% and 57.2%), had an annual household income ≤50,000 yuan (90.5% vs. 83.7% and 85.9%), and lived in rural areas (100.0% vs. 52.0% and 46.8%).

The subjects at high risk for CVD in the HeXi region were more likely to be overweight and obese (73.1% vs. 65.8% and 61.3%), to have central obesity (52.7% vs. 45.9% and 43.8%), and to be snorers (32.3% vs. 24.6% and 30.6%) than in other regions. The proportion of smokers was greater in the LongDong region than in other regions (30.0% vs. 28.1% and 25.8%), and the proportion of drinkers was greater in the LongZhong region than in other regions (4.2% vs. 3.9% and 2.7%). These characteristics were also observed between disease history and health measurement indicators among subjects at high risk for CVD in all three regions (*P* < 0.001) (Table 1).

**Table 1:** Basic characteristics of subjects with high risk for CVD in Gansu Province from 2017 to 2022 [*n* (%)].

Characteristics	All (*N* = 21,059)	HeXi region (*N* = 6,203)	LongZhong region (*N* = 7,554)	LongDong region (*N* = 7,302)	*F*/*χ*^2^	*P-*value*
Age (years, $\bar{x}\pm s$)	58.4 ± 9.3	59.0 ± 9.3	58.8 ± 9.6	57.5 ± 9.0	122.1	**<0**.**001**
Age (years)					192.2	**<0**.**001**
35–39	570 (2.7)	168 (2.7)	208 (2.8)	194 (2.7)		
40–49	3,241 (15.4)	835 (13.5)	1,177 (15.6)	1,229 (16.8)		
50–59	7,283 (34.6)	2,043 (32.9)	2,416 (32.0)	2,824 (38.7)		
60–69	7,206 (34.2)	2,294 (37.0)	2,590 (34.3)	2,322 (31.8)		
70–75	2,759 (13.1)	863 (13.9)	1,163 (15.4)	733 (10.0)		
Gender					25.0	**<0**.**001**
Male	10,538 (50.0)	3,253 (52.4)	3,770 (49.9)	3,515 (48.1)		
Female	10,521 (50.0)	2,950 (47.6)	3,784 (50.1)	3,787 (51.9)		
Educational attainment					796.0	**<0**.**001**
Primary school and below	12,088 (57.4)	2,803 (45.2)	4,322 (57.2)	4,963 (68.0)		
Junior high school	4,720 (22.4)	1,816 (29.3)	1,538 (20.4)	1,366 (18.7)		
High school/technical secondary school	2,686 (12.8)	972 (15.7)	1,123 (14.9)	591 (8.1)		
College degree or above	1,525 (7.2)	607 (9.8)	550 (7.3)	368 (5.0)		
Unclear	40 (0.2)	5 (0.1)	21 (0.3)	14 (0.2)		
Occupation					1,400.0	**<0**.**001**
Farmer	12,971 (61.6)	2,847 (45.9)	4,551 (60.3)	5,573 (76.3)		
Non-farmer	7,905 (37.5)	3,320 (53.5)	2,919 (38.6)	1,666 (22.8)		
Unclear	183 (0.9)	36 (0.6)	84 (1.1)	63 (0.9)		
Areas					5,600.0	**<0**.**001**
Urban	6,991 (33.2)	2,976 (48.0)	4,015 (53.2)	-		
Rural	14,068 (66.8)	3,227 (52.0)	3,539 (46.8)	7,302 (100.0)		
Annual household income (yuan)					322.5	**<0**.**001**
>50,000	1,979 (9.4)	856 (13.8)	643 (8.5)	480 (6.6)		
≤50,000	18,292 (86.9)	5,193 (83.7)	6,490 (85.9)	6,609 (90.5)		
Unclear	788 (3.7)	154 (2.5)	421 (5.6)	213 (2.9)		
Disease history (yes)						
Hypertension	16,984 (80.7)	4,807 (77.5)	6,162 (81.6)	6,015 (82.4)	57.6	**<0**.**001**
Diabetes	4,599 (21.8)	1,502 (24.2)	1,671 (22.1)	1,426 (19.5)	43.7	**<0**.**001**
Dyslipidemia	4,478 (21.3)	1,635 (26.4)	1,450 (19.2)	1,393 (19.1)	136.3	**<0**.**001**
Self-reported cardiovascular and cerebrovascular history	3,629 (17.2)	1,747 (28.2)	768 (10.2)	1,114 (15.3)	804.1	**<0**.**001**
Smoking					32.4	**<0**.**001**
Yes	5,883 (27.9)	1,745 (28.1)	1,949 (25.8)	2,189 (30.0)		
No	15,176 (72.1)	4,458 (71.9)	5,605 (74.2)	5,113 (70.0)		
Drinking					24.9	**<0**.**001**
Yes	753 (3.6)	241 (3.9)	314 (4.2)	198 (2.7)		
No	20,306 (96.4)	5,962 (96.1)	7,240 (95.8)	7,104 (97.3)		
Snoring					196.8	**<0**.**001**
Yes	6,100 (29.0)	2,003 (32.3)	1,859 (24.6)	2,238 (30.6)		
No	5,199 (24.7)	1,475 (23.8)	2,112 (28.0)	1,612 (22.1)		
Uncertain	706 (3.3)	303 (4.9)	260 (3.4)	143 (2.0)		
Unclear	9,054 (43.0)	2,422 (39.0)	3,323 (44.0)	3,309 (45.3)		
Overweight and obesity					214.0	**<0**.**001**
Yes	13,986 (66.4)	4,539 (73.2)	4,971 (65.8)	4,476 (61.3)		
No	7,073 (33.6)	1,664 (26.8)	2,583 (34.2)	2,826 (38.7)		
Central obesity					113.5	**<0**.**001**
Yes	9,941 (47.2)	3,270 (52.7)	3,469 (45.9)	3,202 (43.8)		
No	11,118 (52.8)	2,933 (47.3)	4,085 (54.1)	4,100 (56.2)		
Health measurement indicators						
SBP (mmHg, $\bar{x}\pm s$)	153.7 ± 23.6	149.6 ± 23.8	154.8 ± 23.3	156.1 ± 23.2	292.8	**<0**.**001**
DBP (mmHg, $\bar{x}\pm s$)	89.4 ± 13.5	86.9 ± 13.2	89.6 ± 13.7	91.3 ± 13.3	349.4	**<0**.**001**
GLU (mmol/L, $\bar{x}\pm s$)	6.4 ± 2.0	6.4 ± 2.0	6.5 ± 2.1	6.3 ± 1.9	82.8	**<0**.**001**
BMI (kg/m^2^, $\bar{x}\pm s$)	25.5 ± 3.4	26.0 ± 3.3	25.5 ± 3.5	25.1 ± 3.5	263.3	**<0**.**001**
WC (cm, $\bar{x}\pm s$)	86.3 ± 9.8	87.8 ± 9.8	86.0 ± 9.8	85.4 ± 9.6	225.3	**<0**.**001**
TG (mmol/L, $\bar{x}\pm s$)	1.9 ± 1.1	2.1 ± 1.2	1.8 ± 1.1	1.9 ± 1.1	340.8	**<0**.**001**
TC (mmol/L, $\bar{x}\pm s$)	4.0 ± 1.2	4.1 ± 1.2	4.0 ± 1.3	3.9 ± 1.1	68.0	**<0**.**001**
HDL-C (mmol/L, $\bar{x}\pm s$)	1.2 ± 0.5	1.1 ± 0.4	1.2 ± 0.4	1.2 ± 0.5	146.1	**<0**.**001**
LDL-C (mmol/L, $\bar{x}\pm s$)	2.2 ± 1.1	2.2 ± 1.0	2.3 ± 1.3	2.2 ± 1.1	62.5	**<0**.**001**

CVD, cardiovascular disease; SBP, systolic blood pressure; DBP, diastolic blood pressure; GLU, blood glucose; BMI, body mass index; WC, waist circumference; TG, triglyceride; TC, total cholesterol; HDL-C, high-density lipoprotein cholesterol; LDL-C, low-density lipoprotein cholesterol.

The bold values indicate *P* < 0.05.

* *P*-values were calculated by using *χ*^2^ tests for categorical variables and Wilcoxon rank-sum tests for continuous variables among region groups from 2017 to 2022.

### 3.2 The prevalence rates of four high-risk types among subjects at high risk for CVD

A total of 21,059 subjects at high-risk for CVD were identified, and the standardized prevalence rate was 19.7%. The prevalence rates were 14.0%, 58.2%, 34.9%, and 5.7% for cardiovascular history, hypertension, dyslipidemia, and WHO-assessed risk ≥20%, respectively (Table 2).

**Table 2:** The prevalence rates of the four high-risk types among subjects at high risk for CVD with different characteristics in Gansu province from 2017 to 2022 [*n* (%)].

Characteristics	All screened subjects	All high-risk subjects	Cardiovascular history type	Hypertension type	Dyslipidemia type	WHO-assessed risk ≥20% type
Total	100,082	21,059 (19.7)	3,629 (14.0)	12,847 (58.2)	6,222 (34.9)	1,843 (5.7)
Age (years)						
35–39	5,506	570 (11.1)	28 (5.4)	237 (43.3)	588 (21.5)	0 (0.0)
40–49	24,198	3,241 (14.2)	278 (8.5)	1,761 (55.4)	1,712 (24.2)	54 (1.6)
50–59	34,269	7,283 (21.5)	1,108 (15.3)	4,582 (62.9)	2,184 (30.3)	298 (4.1)
60–69	26,701	7,206 (27.0)	1,604 (22.5)	4,517 (62.7)	1,406 (41.9)	956 (13.2)
70–75	9,408	2,759 (29.3)	611 (22.3)	1,750 (63.4)	332 (55.6)	535 (19.5)
*χ*^2^		2,200.0	426.0	177.5	730.1	1,000.0
*P*-value*		**<0.001**	**<0.001**	**<0.001**	**<0.001**	**<0.001**
Gender						
Male	44,920	10,538 (22.1)	2,005 (15.3)	5,508 (50.3)	3,929 (42.8)	916 (5.9)
Female	55,162	10,521 (17.3)	1,624 (12.7)	7,339 (66.3)	2,293 (26.7)	927 (5.5)
*χ*^2^		286.7	47.6	676.8	606.8	0.1
*P-*value*		**<0.001**	**<0.001**	**<0.001**	**<0.001**	0.761
Educational attainment						
Primary school and below	55,736	12,088 (18.7)	1,938 (12.3)	8,184 (62.1)	2,906 (33.3)	1,255 (6.1)
Junior high school	23,138	4,720 (19.8)	884 (15.9)	2,579 (54.8)	1,626 (36.0)	350 (5.6)
High school/technical secondary school	12,501	2,686 (20.1)	552 (16.6)	1,374 (52.7)	978 (38.0)	173 (4.8)
College degree or above	8,528	1,525 (20.8)	250 (18.7)	683 (46.6)	699 (41.1)	61 (4.7)
Unclear	179	40 (18.5)	5 (12.0)	27 (52.1)	13 (38.5)	4 (2.7)
*χ*^2^		72.7	41.7	587.3	485.9	112.0
*P*-value*		**<0.001**	**<0.001**	**<0.001**	**<0.001**	**<0.001**
Occupation						
Farmer	61,928	12,971 (19.2)	1,939 (12.3)	8,781 (63.3)	3,255 (31.7)	1,205 (6.0)
Non-farmer	37,199	7,905 (20.5)	1,654 (16.8)	3,991 (50.3)	2,871 (39.8)	625 (5.2)
Unclear	955	183 (18.9)	36 (15.8)	75 (33.2)	96 (61.8)	13 (4.6)
*χ*^2^		3.3	123.7	642.6	343.8	12.4
*P-*value*		0.187	**<0.001**	**<0.001**	**<0.001**	**0.002**
Areas						
Urban	33,746	6,991 (19.1)	1,347 (16.1)	3,751 (51.5)	2,356 (37.9)	550 (4.7)
Rural	66,336	14,068 (20.1)	2,282 (13.1)	9,096 (61.4)	3,866 (33.3)	1,293 (6.2)
*χ*^2^		3.2	30.4	237.7	86.8	10.2
*P*-value*		0.072	**<0.001**	**<0.001**	**<0.001**	**0.001**
Annual household income (yuan)						
>50,000	9,826	1,979 (20.6)	411 (18.1)	928 (47.4)	785 (40.9)	123 (5.1)
≤50,000	86,400	18,292 (19.6)	3,113 (13.8)	11,439 (59.8)	5,165 (33.6)	1,638 (5.7)
Unclear	3,856	788 (19.2)	105 (10.4)	480 (53.4)	272 (44.1)	82 (5.6)
*χ*^2^		6.5	26.4	183.7	121.8	19.6
*P*-value*		**0.038**	**<0.001**	**<0.001**	**<0.001**	**<0.001**
Smoking						
Yes	24,194	5,883 (22.9)	1,050 (12.4)	3,079 (51.0)	2,229 (43.9)	635 (9.8)
No	75,888	15,176 (19.0)	2,579 (14.4)	9,768 (58.6)	3,993 (34.3)	1,208 (4.7)
*χ*^2^		205.9	2.2	257.8	273.0	42.6
*P*-value*		**<0.001**	0.141	**<0.001**	**<0.001**	**<0.001**
Drinking						
Yes	3,102	753 (21.4)	109 (14.6)	468 (63.6)	216 (25.7)	86 (6.8)
No	96,980	20,306 (19.7)	3,520 (14.1)	12,379 (57.7)	6,006 (35.3)	1,757 (5.6)
*χ*^2^		20.14	4.2	0.4	0.3	7.0
*P*-value*		**<0.001**	**0.041**	0.511	0.598	**0.008**
Snoring						
Yes	23,180	6,100 (24.6)	1,334 (17.6)	3,434 (55.4)	1,873 (34.5)	464 (5.0)
No	30,150	5,199 (16.9)	779 (12.4)	3,293 (57.9)	1,477 (35.7)	406 (4.7)
Uncertain	3,349	706 (20.4)	122 (13.2)	380 (52.0)	250 (40.3)	43 (3.2)
Unclear	43,403	9,054 (19.1)	1,394 (12.7)	5,740 (61.2)	2,622 (33.3)	930 (6.9)
*χ*^2^		646.1	88.7	66.6	17.5	2.6
*P*-value*		**<0.001**	**<0.001**	**<0.001**	**<0.001**	0.271
BMI (kg/m^2^)						
<18.5	2,021	238 (10.0)	43 (11.4)	140 (52.1)	70 (41.8)	14 (2.5)
18.5–	44,637	6,835 (14.1)	1,262 (15.2)	4,168 (56.3)	1,818 (34.0)	599 (5.3)
24.0–	39,164	9,267 (22.2)	1,605 (14.0)	5,518 (57.4)	2,875 (35.6)	814 (5.9)
≥28.0	14,260	4,719 (31.5)	719 (12.4)	3,021 (62.5)	1,459 (34.5)	416 (6.1)
*χ*^2^		2,400.0	20.6	26.8	42.5	2.5
*P*-value*		**<0.001**	**<0.001**	**<0.001**	**<0.001**	0.477
Central obesity						
Yes	34,469	9,941 (27.0)	1,623 (13.1)	6,147 (59.9)	3,083 (35.5)	944 (6.4)
No	65,613	11,118 (16.0)	2,006 (14.7)	6,700 (56.7)	3,139 (34.4)	899 (5.1)
*χ*^2^		1,900.0	10.8	5.4	19.5	13.1
*P*-value*		**<0.001**	**0.001**	**0.020**	**<0.001**	**<0.001**
History of hypertension						
Yes	41,241	16,984 (40.0)	2,546 (11.6)	12,847 (77.3)	3,153 (20.1)	1,842 (6.9)
No	58,841	4,075 (7.2)	1,083 (24.4)	0 (0.0)	3,069 (77.1)	1 (0.1)
*χ*^2^		17,000.0	309.3	7,900.0	5,100.0	481.9
*P*-value*		**<0.001**	**<0.001**	**<0.001**	**<0.001**	**<0.001**
History of diabetes						
Yes	14,286	4,599 (29.7)	829 (13.7)	2,786 (61.1)	1,385 (33.6)	702 (9.2)
No	85,796	16,460 (18.3)	2,800 (14.0)	10,061 (57.8)	4,837 (35.0)	1,141 (4.7)
*χ*^2^		1,200.0	2.6	0.4	0.9	312.5
*P*-value*		**<0.001**	0.107	0.502	0.338	**<0.001**
History of dyslipidemia						
Yes	11,259	4,478 (37.8)	626 (11.8)	1,921 (40.3)	2,561 (60.2)	456 (7.7)
No	88,823	16,581 (17.4)	3,003 (14.6)	10,926 (63.4)	3,661 (27.6)	1,387 (5.2)
*χ*^2^		2,700.0	42.2	783.8	2,100.0	14.6
*P*-value*		**<0.001**	**<0.001**	**<0.001**	**<0.001**	**<0.001**
Self-reported history of cardiovascular and cerebrovascular conditions						
Yes	3,629	3,629 (100.0)	3,629 (100.0)	578 (13.7)	266 (7.0)	112 (1.6)
No	96,453	17,430 (17.3)	0 (0.0)	12,269 (65.8)	5,956 (38.9)	1,731 (6.7)
*χ*^2^		14,000.0	21,000.0	3,700.0	1,000.0	176.2
*P*-value*		**<0.001**	**<0.001**	**<0.001**	**<0.001**	**<0.001**

CVD, cardiovascular disease; BMI, body mass index.

The bold values indicate *P* < 0.05.

* *P*-values were calculated by using *χ*^2^ tests for different characteristics of groups for the overall and four high-risk types from 2017 to 2022.

**Figure 2:**
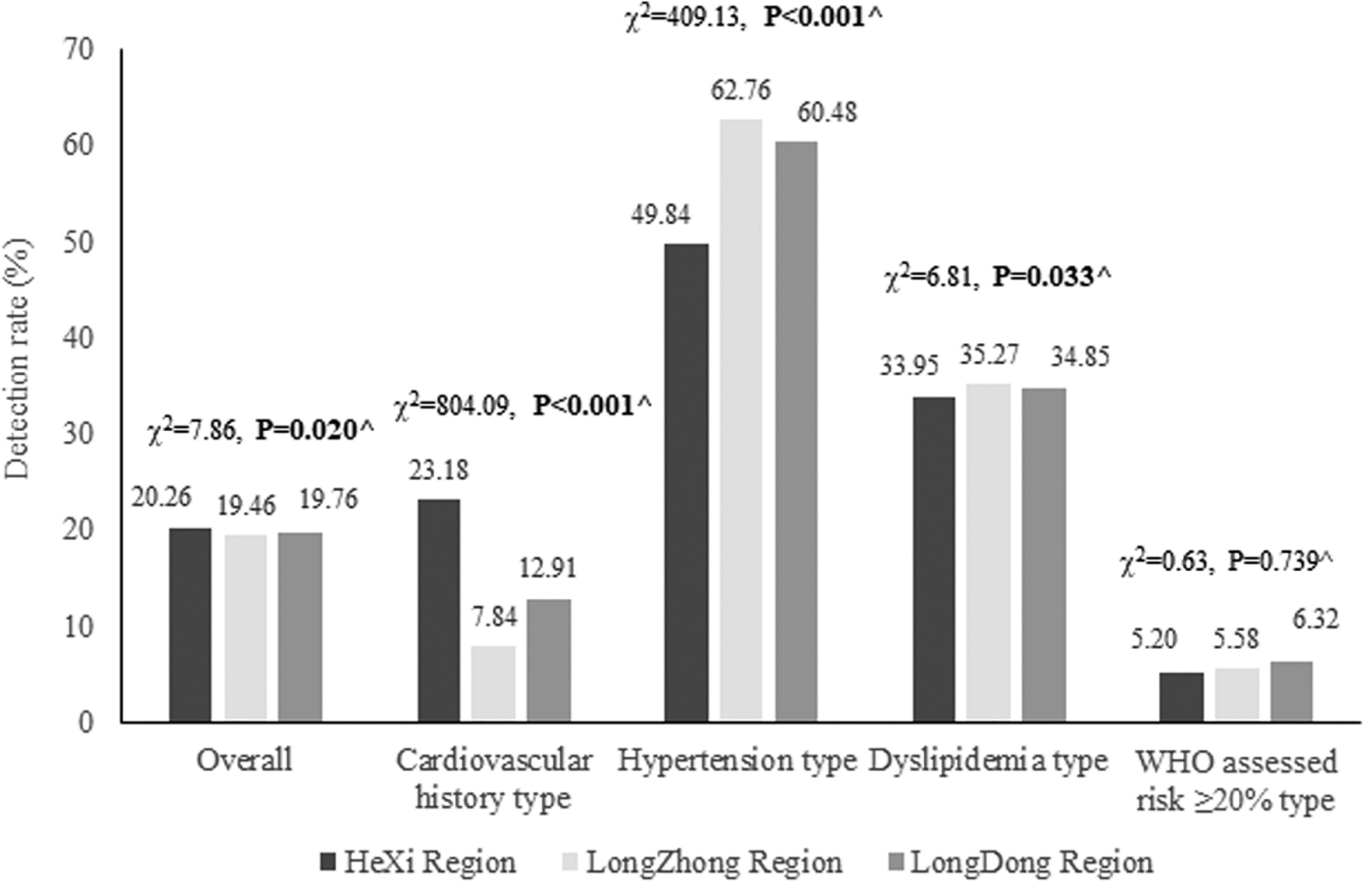
The prevalence rates of four high-risk types among the subjects at high risk for CVD in different regions of Gansu Province from 2017 to 2022. CVD, cardiovascular disease. Total screening subjects in the HeXi, LongZhong, and LongDong regions were 28,711, 36,182, and 35,189, respectively. Total subjects at high risk for CVD in the HeXi, LongZhong, and LongDong regions were 6,203, 7,554, and 7,302, respectively. ^*P*-values were calculated by using *χ*^2^ tests for overall and the four high-risk types in different regions from 2017 to 2022.

The overall prevalence rate of subjects at high risk for CVD in the HeXi region was greater than that in other regions, and the prevalence rate of cardiovascular history type was also the same (*P* < 0.05). However, the prevalence rates of hypertension and dyslipidemia were the highest in the LongZhong region (*P* < 0.05), and the prevalence rate of WHO-assessed risk ≥20% was the highest in the LongDong region (Figure 2).

Table 2 shows the differences in the prevalence rates of subjects at high risk for CVD with different characteristics. The prevalence rates of subjects at high risk for CVD among elderly subjects, males, non-farmers, smokers, drinkers, snorers, subjects with college degree or above, subjects with an annual household income >50,000 yuan, subjects living in rural areas, subjects with obesity, and subjects with a history of disease were greater than those in other groups (*P* < 0.05). Similar results were also observed in the prevalence rates of the four high-risk types for different subgroups, including cardiovascular history, hypertension, dyslipidemia, and WHO-assessed risk ≥20% (*P* < 0.05) (Table 2).

### 3.3 Risk factors associated with subjects at high risk for CVD in different regions

Table 3 indicates that in the HeXi and LongDong regions, with increasing age, the risk of becoming a subject at high risk for CVD increased (*P* < 0.001), while 40–49 years of age was a protective factor for high risk of CVD in the LongZhong region (OR: 0.8, 95% CI: 0.7–1.0). Male, smoking status, overweight and obesity status, and disease history were positively associated with high risk of CVD (all *P* < 0.05). Snorers and central obesity were positively associated with high risk of CVD in the HeXi and LongZhong regions but not in the LongDong region (all *P* < 0.05).

**Table 3:** Mixed effects model analysis for risk factors associated with subjects at high risk for CVD.

Characteristics	HeXi region		LongZhong region		LongDong region	
	OR (95% CI)	*P*-values*	OR (95% CI)	*P*-values*	OR (95% CI)	*P*-values*
Age (years) (ref: 35–39)						
40–49	1.0 (0.8, 1.2)	0.926	0.8 (0.7, 1.0)	**0**.**026**	1.1 (0.9, 1.3)	0.234
50–59	1.4 (1.1, 1.7)	**0**.**001**	0.9 (0.8, 1.1)	0.537	1.4 (1.1, 1.6)	**<0**.**001**
60–69	1.8 (1.5, 2.2)	**<0**.**001**	1.0 (0.9, 1.2)	0.759	1.6 (1.3, 1.9)	**<0**.**001**
70–75	2.0 (1.6, 2.4)	**<0**.**001**	1.0 (0.9, 1.2)	0.705	1.8 (1.4, 2.1)	**<0**.**001**
Gender (ref: female)						
Male	1.6 (1.3, 2.0)	**<0**.**001**	1.4 (1.2, 1.6)	**<0**.**001**	1.3 (1.1, 1.5)	**<0**.**001**
Educational attainment (ref: primary school and below)						
Junior high school	1.0 (0.8, 1.2)	0.891	0.9 (0.8, 1.0)	0.069	1.1 (0.9, 1.2)	0.395
High school/technical secondary school	1.0 (0.8, 1.3)	0.748	0.8 (0.7, 1.0)	0.072	1.0 (0.9, 1.3)	0.622
College degree or above	1.1 (0.8, 1.5)	0.684	1.3 (1.0, 1.6)	**0**.**049**	0.9 (0.7, 1.2)	0.670
Occupation (ref: non-farmer)						
Farmer	1.6 (1.3, 1.9)	**<0**.**001**	1.0 (0.8, 1.1)	0.741	0.8 (0.7, 0.9)	**<0**.**001**
Areas (ref: Rural)						
Urban	1.4 (1.2, 1.7)	**<0**.**001**	0.6 (0.6, 0.7)	**<0**.**001**	–	–
Annual household income (yuan) (ref: ≤50,000)						
>50, 000	1.1 (0.9, 1.4)	0.255	0.7 (0.6, 0.9)	**0**.**001**	1.3 (1.0, 1.6)	**0**.**046**
Smoking (ref: no)						
Yes	1.6 (1.3, 2.0)	**<0**.**001**	1.2 (1.1, 1.4)	**0**.**002**	1.4 (1.2, 1.6)	**<0**.**001**
Drinking (ref: no)						
Yes	0.8 (0.7, 0.9)	**0**.**006**	0.8 (0.6, 1.0)	0.082	0.8 (0.6, 1.1)	0.162
Snoring (ref: no)						
Yes	1.2 (1.1, 1.3)	**0**.**001**	1.0 (0.9, 1.1)	0.611	1.2 (1.1, 1.3)	**<0**.**001**
BMI (kg/m^2^) (ref: 18.5∼)						
<18.5	0.3 (0.1, 0.8)	**0**.**012**	0.8 (0.6, 1.2)	0.364	0.9 (0.6, 1.3)	0.625
24.0–	1.4 (1.2, 1.7)	**<0**.**001**	1.3 (1.2, 1.5)	**<0**.**001**	1.3 (1.2, 1.5)	**<0**.**001**
≥28.0	1.9 (1.5, 2.4)	**<0**.**001**	1.7 (1.5, 2.0)	**<0**.**001**	1.7 (1.5, 2.1)	**<0**.**001**
Central obesity (ref: no)						
Yes	1.2 (1.0, 1.5)	**0**.**021**	1.1 (1.0, 1.2)	0.187	1.3 (1.2, 1.5)	**<0**.**001**
Disease history (ref: no)						
Hypertension	5.7 (5.3, 6.1)	**<0**.**001**	9.9 (9.2, 10.6)	**<0**.**001**	8.8 (8.3, 9.5)	**<0**.**001**
Diabetes	1.3 (1.1, 1.6)	**0**.**003**	1.2 (1.0, 1.3)	**0**.**005**	1.5 (1.3, 1.7)	**<0**.**001**
Dyslipidemia	8.3 (7.0, 10.0)	**<0**.**001**	7.0 (5.6, 8.8)	**<0**.**001**	4.2 (3.5, 5.2)	**<0**.**001**

CVD, cardiovascular disease; BMI, body mass index.

The bold values indicate *P* < 0.05.

All mixed effects models adjusted for age, gender, occupation, smoking status, drinking status, annual household income, educational attainment, BMI, central obesity, areas, and disease history.

Subjects with college degree or above were positively associated with high risk of CVD in the LongZhong region (OR: 1.3, 95% CI: 1.0–1.6) but not in the HeXi and LongDong regions. Subjects with annual household income >50,000 yuan were negatively associated with high risk of CVD in the LongZhong region (OR: 0.7, 95% CI: 0.6–0.9), but positively in the LongDong region (OR: 1.3, 95% CI: 1.0–1.6). Being a farmer or a subject living in urban areas was a risk factor for being at high risk for CVD in the HeXi region (farmer: OR: 1.6, 95% CI: 1.3–1.9; urban: OR: 1.4, 95% CI: 1.2–1.7); however, being a farmer was a protective factor in the LongDong region (OR: 0.8, 95% CI: 0.7–0.9), and being a subject living in urban areas was a protective factor in the LongZhong region (OR: 0.6, 95% CI: 0.6–0.7). Only drinkers in the HeXi region were negatively associated with high risk of CVD (OR: 0.8, 95% CI: 0.7–0.9).

## 4 Discussion

To our knowledge, this study is the first to examine the prevalence rate of subjects at high risk for CVD and to analyze the influencing factors associated with high-risk subjects of CVD in different regions of Gansu Province from 2017 to 2022. First, the overall prevalence rate of subjects at high risk for CVD was 19.7%, and the prevalence rate in the HeXi region was greater than that in other regions. Second, the prevalence rates were 14.0%, 58.2%, 34.9%, and 5.7% for cardiovascular history, hypertension, dyslipidemia, and WHO-assessed risk ≥20%, respectively. The prevalence rate of cardiovascular history type was the highest in the HeXi region, hypertension and dyslipidemia types were the highest in the LongZhong region, and WHO-assessed risk ≥20% was the highest in the LongDong region. Third, male, higher education level, smoking status, snoring status, overweight and obesity status, central obesity status, and subjects with disease history were risk factors. There were some differences among the different regions in terms of age, annual household income, farmer status, rural/urban status, and drinking status.

The prevalence rate of subjects at high risk for CVD in Gansu Province (19.7%) was lower than that at the national level (26.3%) (16) and that in Jiangsu Province (25.0%) (17), but higher than that in Sichuan (17.6%) (18) and Hainan Provinces (17.7%) (19), which may be related to differences in the selection of screening tools, the unequal distribution of community health resources, the climate and environment, and lifestyles. Gansu Province is in the northwestern region of China, and has relatively scarce health resources (6). The distribution of screening techniques and tools and community health resources was uneven among the different project sites. It is imperative to strengthen the capacity building of medical staff, improve the quality of medical services, and improve early screening coverage. Among the four high-risk types of CVD, the prevalence rate of hypertension was the highest, which was consistent with the findings of the studies of Wu et al. (17), Chang et al. (20), and Yue et al. (21). Hypertension is an independent and important risk factor for CVD, and blood pressure is closely related to the risk of CVD occurrence and death (22). For every 20 mmHg increase in SBP or 10 mmHg increase in DBP, the risk of CVD development doubles (23). If all hypertensive patients were treated, there would be a reduction of 803,000 CVD events per year and 1.2 million health-adjusted life years gained (24). Therefore, the prevention and control of hypertension should be prioritized.

We found that there were differences in the prevalence rates of the four high-risk types among the subjects at high risk for CVD in different regions. The disparity may be mainly due to differences in demographic and socioeconomic characteristics (such as the proportion of farmers, annual household income, and education), geographical features, climatic conditions, endemic diseases, lifestyles, etc. For example, the HeXi region was mainly related to a high proportion of non-farmers, overweight and obese subjects, annual household income >50,000 yuan, drinkers, and subjects with self-reported cardiovascular and cerebrovascular history. However, the LongZhong region was associated with a high proportion of drinkers, overweight and obese subjects, and subjects with a history of hypertension and dyslipidemia. The LongDong region had a high proportion of farmers, smokers, and subjects with a history of hypertension and dyslipidemia. The LongDong and LongZhong regions are dominated by the Loess Plateau, while the HeXi region has a flat terrain (5). The climate in the LongDong region is humid, that in the LongZhong region is semiarid, and that in the HeXi region is arid (5). These reasons may have caused regional differences.

In the present study, the prevalence rate of subjects at high risk for CVD increased with increasing age, which was consistent with the previous studies (18, 19, 25). Increased age was a risk factor in the HeXi and LongDong regions, and younger age was a protective factor in the LongZhong region. With increasing age, physical function continues to deteriorate and damage and various high-risk CVD factors accumulate, overlap, and produce synergistic effects on the body (19). Being of male gender was a risk factor in different regions, and the percentage of male subjects was greater than that of females. This may be related to the lifestyle, diet, and physiological structure of males, who are more likely to be associated with high-risk CVD factors (26).

One of the exceptional findings of our study was that being a farmer and living in urban areas were risk factors for CVD in the HeXi region. Subjects with a college degree or above, annual household income ≤50,000 yuan, and living in rural areas were positively associated with high-risk of CVD in the LongZhong region. Having an annual household income >50,000 yuan and being a non-farmer were risk factors in the LongDong region. The HeXi region has a flat terrain, abundant resources, and developed agricultural, mineral, and tourism resources, and its GDP has always been at the forefront of that of Gansu Province (27). The LongZhong region has obvious geographical advantages, industrial cities and transportation hubs, intensive technology and human resources, and a high level of economic development, highlighting the economic development of Gansu Province (27). The economic development of the LongDong region is dominated by a small-scale peasant economy and natural economy, with backward production technology and a weak industrial foundation (27). For terrain and climate reasons, the farmers in the HeXi region were mainly involved in animal husbandry, and the farmers in the LongDong and LongZhong regions were mainly involved in agriculture. Urban and rural residents in these regions have different eating habits, such as those in the HeXi region, who like to eat beef and mutton (strongly flavored food), and those in the LongDong and LongZhong regions, who like to eat rice, wheat, and other foods (7). Moreover, differences in education level and economic status are closely related to differences in residents’ health status. On the one hand, when people with a high level of education and good economic conditions pay more attention to their own health issues, their understanding and implementation of health knowledge will also increase, medical and health resources will improve, and the demand and utilization of health services will increase (28, 29). On the other hand, socioeconomic status can also increase psychological stress and life pressure, thus affecting the incidence of CVD, such as in urban areas in the HeXi region.

In addition, smoking was a risk factor of subjects at high-risk for CVD (30, 31), which was consistent with our study. Nicotine, the primary ingredient of tobacco, is a sympathetic stimulant that acts on the central and peripheral nervous systems to stimulate the release of catecholamine and other neurotransmitters. It leads to cardiovascular consequences, such as elevated heart rate, blood pressure, and cardiac output (32). However, drinking was negatively associated with high risk of CVD only in the HeXi region. This may be related to the lifestyle of the HeXi region, such as residents indulging in moderate drinking. The relationship between CVD and alcohol intake is complex and responds to hormetic behavior, as reflected by U- or J-shaped relationships, with low–moderate intake being more protective than abstention or abusive drinking (33). We also found that snoring was a risk factor for CVD, but some studies reported that snoring was not associated with increased risk for CVD (34, 35), whereas others reported an increased risk for CVD events (36, 37). The exact mechanisms linking snoring to the development of cardiovascular disorders have not been completely elucidated. Snoring might increase the risk of CVD through atherosclerosis (38) and is also linked to hypertension, diabetes, metabolic syndrome, and obesity, all of which may increase the risk of cardiovascular disorders (39).

Another important finding of this study is that being overweight and obese were positively associated with high risk of CVD, which is in agreement with the findings of other studies (40, 41). This might be because individuals with obesity have greater circulating blood volume, which in turn increases cardiac output, leading to an increased burden on the heart and resulting in a series of CVD events (40). Obesity is not only an independent risk factor for CVD but also closely associated with several other risk factors, such as hypertension and diabetes (41). In this study, subjects with a history of hypertension, diabetes, and dyslipidemia were observed to be at a higher risk for CVD than those without the history, while CVD was also a risk factor for chronic diseases, and various risk factors interact with each other.

Only the Xinjiang Uygur Autonomous Region, Tibet Autonomous Region, and Ningxia Hui Autonomous Region in the western areas of China did this study, and the prevalence rates of subjects at high risk for CVD were 22.1%, 22.1%, and 18.8%, respectively, and other results were similar to those in Gansu Province (42, 43). Interestingly, different provinces in western China have different landforms, climates, and ethnic characteristics, which need to be explored according to different characteristics. Other epidemiologists could consider conducting in-depth research based on terrain, climate, and ethnic characteristics. Similar research has not been conducted in Gansu Province before; therefore, this study identified directions for future research on different regions and populations and could serve as a reference for CVD prevention in Gansu Province, China.

This study has several strengths. First, this study is the first to compare the prevalence and influencing factors for subjects at high risk for CVD in the HeXi, LongDong, and LongZhong regions of Gansu Province. These findings are of great significance to the prevention and treatment of CVD in Gansu Province. Second, the data we used were from the China PEACE MPP with good data quality, and the sample used in our study was large enough to reflect the prevalence rate and influencing factors of high-risk subjects of CVD in Gansu Province to some extent. However, some limitations should be noted. First, the data used are cross-sectional in nature. Thus, causality cannot be inferred. Second, a questionnaire survey was used in this study, which may have led to recall bias. Third, the definitions of several covariates (e.g., smoking and drinking) were defined in two categories (yes/no) without a more detailed definition. These variables were also self-reported, and there was a certain bias in the reliability of the data. Caution is needed when interpreting such results. Fourth, our study did not include data on specific geographic environments, climatic conditions, or endemic diseases, and further studies on the effects of these characteristics on the risk of cardiovascular disease in different regions are needed.

## 5 Conclusion

This study showed that although the prevalence rate of subjects at high risk for CVD was lower than the national level, it is still relatively high in Gansu Province, and the subjects at high risk for CVD in different regions of Gansu Province have different characteristics. Therefore, in the future, we should combine the high-risk strategy with the whole population strategy and take targeted preventive strategies and measures for different regions. We should strengthen publicity and education to promote healthy lifestyles, such as not smoking or drinking, and preventing obesity. We can also identify high-risk subjects through early screening in areas with a high incidence of CVD, especially those who are already at high risk for hypertension or diabetes.

## Data Availability

The raw data supporting the conclusions of this article will be made available by the authors, upon reasonable request, to any qualified researcher.
